# Screening Breast MRI and Genetic Testing Utilization in High-risk Population: Analysis of National Health Interview Survey

**DOI:** 10.1093/jbi/wbaf084

**Published:** 2026-07-28

**Authors:** Ishana Maini, Kevin Gilotra, Maedeh Sharifian, Nina Capiro, Gelareh Sadigh

**Affiliations:** 1 Chicago College of Osteopathic Medicine, Midwestern University, Downers Grove, IL, USA,; 2 Renaissance School of Medicine, Stony Brook University, Stony Brook, NY, USA,; 3 Department of Radiological Sciences, University of California, Irvine, Irvine, CA, USA,; 4 Department of Radiological Sciences, University of California, Los Angeles, Los Angeles, CA, USA

**Keywords:** MRI, screening, genetic testing, breast cancer, high risk

## Abstract

**Objective:**

For individuals at increased risk of breast cancer, additional tests, such as screening breast MRI and genetic testing, are recommended. We assessed the utilization of screening breast MRI and genetic testing among high-risk individuals and identified associated patient factors.

**Methods:**

This retrospective, cross-sectional study used 2023 National Health Interview Survey data. Women aged 25 or older with at least 1 first-degree relative diagnosed with breast cancer before age 50 were included. A survey weight was applied to generate weighted samples representing national estimates. Multivariable logistic regression analysis was used to identify factors associated with screening breast MRI, genetic test discussions, and genetic test completion.

**Results:**

The weighted sample included 6 851 757 women (unweighted *n* = 920) (mean age, 60.2; 3.1% [199 883/6 486 458] Asian, 14.4% [932 502/6 486 458] Black, 78.1% [5 063 194/6 486 458] White, and 4.4% [290 879/6 486 458] other races). Overall, 12.5% (855 463/6 851 757) were of Hispanic ethnicity. Screening MRI use was reported by 7.9% (506 672/6 417 453), while 28.8% (1 963 524/6 822 303) discussed and 17.7% (1 209 406/6 830 229) completed genetic testing. Having 2 or more first-degree relatives with breast cancer increased the likelihood of screening MRI (odds ratio [OR], 2.94; 95% CI, 1.56–5.54) and genetic testing completion (OR, 2.74; 95% CI, 1.68–4.48). The odds of discussing genetic testing were lower for Black individuals (OR, 0.43; 95% CI, 0.20–0.91) and those with Medicaid or no insurance (OR, 0.45; 95% CI, 0.23–0.87). Concern about medical bills was associated with lower odds of genetic testing completion (OR, 0.54; 95% CI, 0.33–0.90).

**Conclusions:**

Screening breast MRI and genetic testing are underutilized among high-risk women. Expanding insurance coverage and provider education may help increase uptake of recommended screening practices.

## Introduction

Breast cancer is one of the most common malignancies world-wide, with more than 2 million cases reported per year and a mortality rate of approximately 2.5%.^1^ Annual screening with mammography has improved breast cancer survival.^2^ Recently, breast MRI has been increasingly used for supplemental screening in patients with an elevated lifetime risk of breast cancer.^3^ Breast MRI is a more sensitive screening modality compared with mammography, breast US, or both tests combined, making it a useful tool for early cancer detection in patients at high risk.^4,5^

The American College of Radiology recommends annual breast MRI as a supplemental screening tool in women with “higher than average risk” of breast cancer.^6^ This group includes women with a history of chest radiation at a young age, a personal or family history of *BRCA* mutations or cancer syndromes, and those with >20% to 25% lifetime risk of breast cancer as determined by Tyrer Cuzick or Gail risk assessment tools.^6–10^ Of women with a family history of breast cancer, those with first-degree relatives diagnosed before age 50 are more likely to have elevated risk when compared with those with relatives diagnosed after age 50, and based on Tyrer Cuzick risk assessment, have >20% lifetime risk of breast cancer.^9,11,12^

Approximately 9% of adult women in the United States are classified as “higher than average risk” and eligible for screening breast MRI.^13^ Despite well-established literature and societal recommendations supporting the benefits of screening breast MRI, utilization among high-risk individuals remains below 10%, with less than half of primary care providers recommending screening breast MRI to eligible women.^13–15^ Genetic testing, also recommended for individuals at a high risk for breast cancer is similarly underutilized. One study found that only 15% of eligible individuals had undergone genetic testing.^16–18^ The underutilization of both screening breast MRI and genetic testing might be due to several barriers, including limited access, insufficient patient knowledge, low perceived benefits, poor health literacy, lack of provider awareness or agreement with guideline recommendations, insurance coverage limitations, and broader health-related social needs.^18–20^

To our knowledge, no recent nationally representative study has evaluated the use of screening breast MRI and genetic testing. Therefore, we assessed the utilization of screening breast MRI and genetic testing among women with a family history of breast cancer in relatives diagnosed before age 50 as a proxy for the high-risk population, using a nationally representative database. We further identified patient factors associated with utilization.

## Methods

### Human ethics and consent to participate

This publicly available survey study did not use any private identifiable information and thus did not constitute human participant research requiring institutional review board (IRB) oversight. Given the study is not considered human participant research, no consent was needed. An administrative Exempt Self-determination was filed with our institutional IRB (IRB Number 6702).

### Study design and data collection

We performed a cross-sectional analysis of data from the National Health Interview Survey (NHIS) 2023 database. The NHIS survey collects data on a broad range of topics to monitor the health of the U.S. population and provide insight into health across varying demographics. The NHIS adult interview sample population consists of noninstitutionalized civilians aged 18 and above residing within the 50 states and the District of Columbia at the time of the interview.^21^ Excluded from the interview are individuals without a fixed household address, active duty military personnel and civilians living on military bases, persons in long-term care institutions or correctional facilities, and U.S. nationals living in foreign countries.^21^ Participants were selected from more than 300 address clusters located in well-defined geographic areas, with approximately 1 to 2 geographic areas selected per state; the response rate was 40.7%. The NHIS survey responses were primarily collected through in-person interviews with the assistance of a computer and were conducted by U.S. Census Bureau–trained staff following procedures developed by experts from the National Center for Health Statistics (NCHS). All NHIS survey interviews were performed either one-on-one or with the entire household.^21^

### Study population

**Figure 1.**
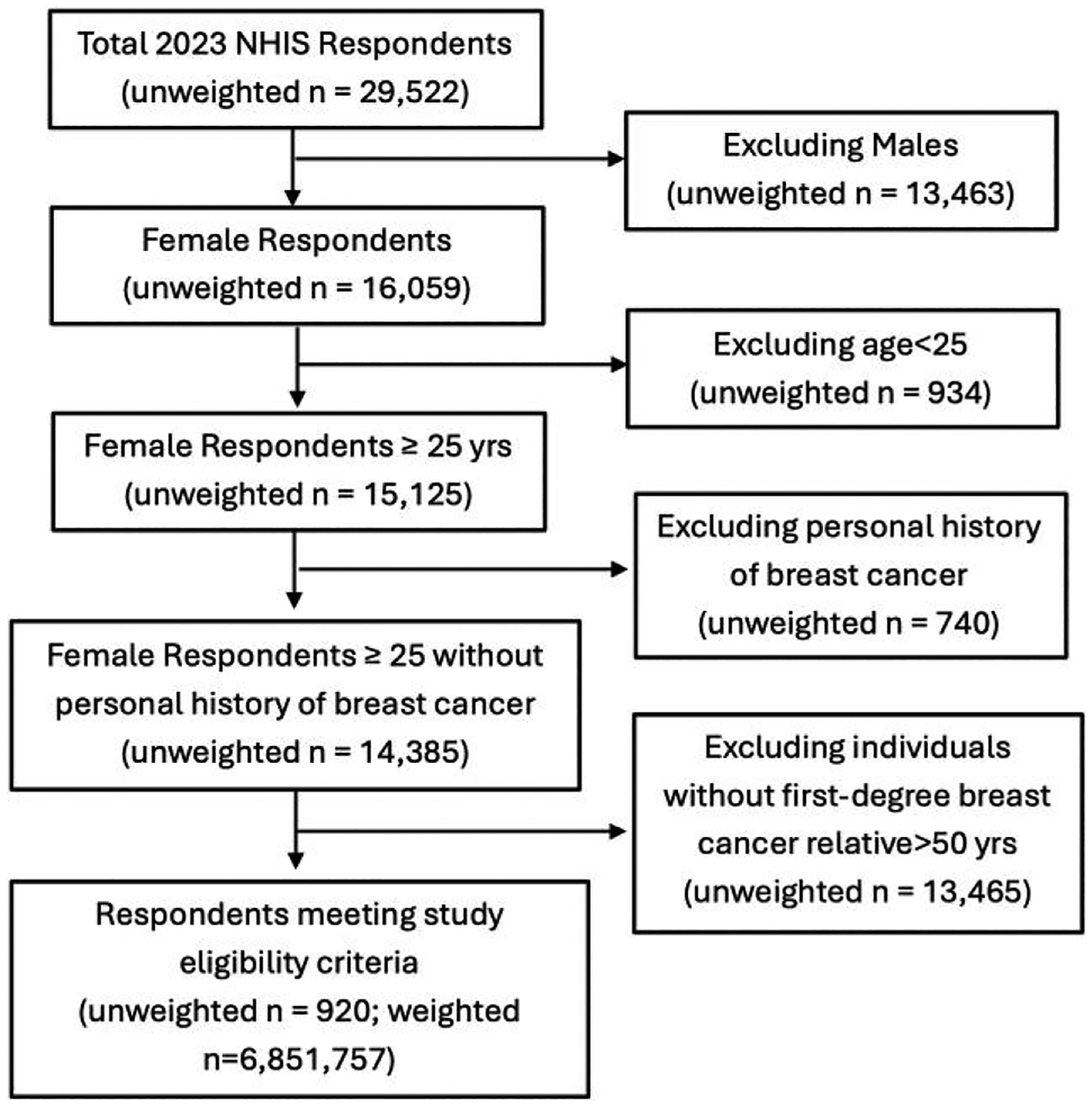
Flow diagram of study inclusion criteria. *n* represents unweighted frequency.

Of the NHIS 2023 sample, female participants 25 or older with at least 1 first-degree relative diagnosed with breast cancer at age 50 or younger were included. Participants with a personal history of breast cancer were excluded. A family history of breast cancer in a first-degree relative, including parents, siblings, or children, diagnosed at or before age 50 was chosen as a proxy for identifying individuals at potentially high risk (Figure 1).

A minimum age of 25 was chosen in this study because the American College of Radiology (ACR) suggests that risk assessment for breast cancer should take place by the age of 25 for all women to allow for supplemental screening for each individual, which may include genetic counseling and, if required, genetic testing. The ACR also recommends that, in women with a ≥20% lifetime risk of breast cancer, annual breast MRI should be started at age 25 to 30.^6,22^ In the NHIS 2023, patients aged 25 and above were queried about use of genetic testing, and women aged 30 and above were surveyed about use of screening mammography and breast MRI.

### Measures

Study outcomes, including receipt of screening breast MRI, genetic testing, or discussions with providers about the possibility of genetic testing, were collected. Breast MRI receipt was assessed via the yes-no survey question “Have you ever had a breast MRI?” and followed by a question inquiring about the main reason for the MRI. Responses of “I am high risk due to family history, genetic test, or another reason” were used to identify screening breast MRIs. Genetic testing was collected through the yes-no question, “Have you ever had a genetic test to determine if you are at greater risk of developing cancer in the future?” Participants were also asked “Have you ever discussed the possibility of getting a genetic test for cancer risk with a doctor or other health care professional?” to capture any discussions regarding genetic testing. Furthermore, information regarding the utilization of screening mammography was also recorded.

Demographic data, such as age, race, ethnicity, language spoken at home, health insurance type, marital status, education, and employment status, were collected. The 2013 NCHS urban-rural classification scheme for counties was used to determine whether individuals lived in nonmetropolitan or metropolitan regions of various sizes.^23^ Household income was recorded as a ratio of family income to the poverty threshold and categorized as 1 of the following: 0 to 1.00, 1.01 to 2.00, and >2.00.^24,25^ Financial hardship as a barrier was assessed by inquiring about anxiety about paying medical bills in the setting of a health concern or major life event. The frequency of Internet access to communicate with physicians’ offices and the time of their most recent doctor’s visit were also documented. The presence of comorbidities and the number of family members with breast cancer were recorded.

#### Statistical analysis

The Final Annual Weight variable, provided within the NHIS survey data by the NCHS, was applied in the STATA software package (Stata/MP 17.0 for Windows, StataCorp, College Station, TX) using svy commands to generate national estimates based on the NHIS, as is best practice to account for sampling probabilities and nonresponse.^21^ Given NHIS is a sample survey, meaning only a subset of the population is selected to participate in the survey, and not everyone selected agrees to participate, the representativeness of the sample can be affected. To account for these 2 factors, sampling weights are created to produce representative national sample estimates.^21^ The weights are generated using a base weight, which is inverse to the probability of selection, and then adjusted for nonresponse patterns (ie, different response rates among different household and person-level subgroups).^21^

Descriptive statistics were used to summarize the sample’s sociodemographic and clinical characteristics and study outcomes. We further conducted a sensitivity analysis, reporting screening MRI utilization among women 30 years or older.

To determine the sociodemographic and clinical characteristics associated with screening breast MRI and genetic testing, we used weighted univariable logistic regression models. A weighted multivariable logistic regression model was constructed. Independent variables included in the model were those that were statistically significant in the univariable model, shown to be associated with the outcome, or were expected to be associated with the outcome. All data analysis was conducted using the STATA software package (Stata/MP 17.0 for Windows, StataCorp, College Station, TX). Statistical significance was defined as a *P*-value <.05.

## Results

### Study population

The total unweighted sample size of NHIS 2023 was 29 522 individuals. Excluding unweighted sample of 13 463 men, 934 individuals aged <25 740 individuals with personal history of breast cancer, and 13 465 individuals without a family history of breast cancer in a first-degree relative before age of 50, the unweighted sample size for current study included 920 women, representing a total weighted sample of 6 851 757 (Figure 1). The mean age was 60.2 years, ranging between the ages of 25 to 97 years. Racial background included 3.1% (199 883/6 486 458) Asian, 14.4% (932 502/6 486 458) Black, 78.1% (5 063 194/6 486 458) White, and 4.4% (290 879/6 486 458) other races, and 12.5% (855 463/6 851 757) were of Hispanic or Latino ethnicity. Baseline characteristics are summarized in Table 1.

### Utilization rate of screening breast MRI and genetic testing

Women aged 30 and above (comprising 94.5% of our weighted population) were asked about prior receipt of a breast MRI. A total of 15.2% reported prior receipt of breast MRI for any reason, and 7.9% reported receipt of a screening breast MRI. However, only 3.6% had a screening breast MRI performed within the last year.

Additionally, 28.8% reported discussions about possibly receiving genetic testing with their providers, while 17.7% reported completing a genetic test. Screening mammography, however, was performed in 77.8% of the population, with 62.5% of these patients receiving it within the last 2 years (Table 2).

### Factors associated with receipt of screening breast MRI

Table 3 highlights the univariable and multivariable analysis of the factors associated with receipt of any screening breast MRI. In multivariate analysis, a higher likelihood of receiving screening breast MRI was associated with using the Internet to communicate with doctors’ offices (odds ratio [OR], 3.02; 95% CI, 1.53–6.00; *P* = .002) and having 2 or more family members with breast cancer (OR, 2.94; 95% CI, 1.56–5.54; *P* = .001).

### Factors associated with receipt of genetic testing

Table 4 highlights the univariable and multivariable analysis of the factors associated with patients discussing the possibility of receiving genetic testing with their health care providers or receiving any genetic testing. On multivariate analysis, age greater than 40, was associated with a lower likelihood of receipt of genetic testing or discussing it with a provider compared with the age group of 25 to 40 years old (*P* < .01). Being married or living with a partner was significantly associated with a higher likelihood of discussing genetic testing (OR, 1.71; 95% CI, 1.14–2.56; *P* = .01) but not with receiving a genetic test. Patients who were Black vs white (OR, 0.43; 95% CI, 0.20–0.91; *P* = .03) or were uninsured or had Medicaid insurance vs commercial insurance (OR, 0.45; 95% CI, 0.23–0.87; *P* = .02) were less likely to discuss genetic testing. Additionally, having 2 or more family members with a history of breast cancer was associated with a higher likelihood of both discussing genetic testing (OR, 2.62; 95% CI, 1.69–4. 05; *P*< .001) and receiving the genetic test (OR, 2.74; 95% CI, 1.68–4.48; *P* < .001).

Patients who used the Internet to communicate with the doctor’s office were significantly more likely to discuss (OR, 2.34; 95% CI, 1.51–3.62; *P* < .001) and receive genetic testing (OR, 2.49; 95% CI, 1.48–4.20; *P* = .001) when compared with patients who did not use the Internet. Prior receipt of screening MRI was also associated with higher likelihood of discussion (OR, 5.60; 95% CI, 3.00–10.45; *P* < .001) and receipt of genetic testing (OR, 4.04; 95% CI, 2.00–8.16; *P* < .001). Compared with patients who visited their doctor’s office within the last 2 years, those who last saw their doctor more than 2 years ago were significantly less likely to discuss genetic testing with their physician (OR, 0.18; 95% CI, 0.05–0.68; *P* = .01). Those worried about potential medical bills also exhibited a decreased likelihood of receiving a genetic test (OR, 0.54; 95% CI, 0.33–0.90; *P* = .02).

## Discussion

In this analysis of the 2023 NHIS, the utilization of screening breast MRI at any time among women with a family history of breast cancer in a first-degree relative diagnosed aged 50 or younger was 7.9%, with only 3.6% completing this within the last year. Furthermore, genetic testing utilization was 17.7% among this group of high-risk women, suggesting underutilization of both tests, despite recommendations by national guidelines.^6,26^

Our study shows persistent underutilization of screening breast MRI in high-risk women. Studies estimate that 5.8% to 9% of all women between the ages of 25 and 64 have an elevated lifetime breast cancer risk and are therefore eligible for screening breast MRI.^13,27^ However, a 2010 analysis of NHIS data showed approximately 10% of women with increased lifetime risk of breast cancer reported ever having had a breast MRI for screening or diagnostic purposes^28,29^ Another study showed screening breast MRI utilization ranging from 2% among women with family history of breast cancer to 20% among women with *BRCA* mutation.^29^

The underutilization of screening breast MRI is likely due to factors at the patient and provider levels. Providers may not be aware of the current high-risk breast cancer screening guidelines and thus may not recommend screening breast MRI to eligible patients.^15^ For example, 1 study found that less than half of providers recommended screening breast MRI for high-risk women.^15^ Providers may also disagree with guideline recommendations, especially because early and repeat screening MRIs have been shown to increase the likelihood of false-positive tests, benign biopsies, and patient anxiety.^30^ Due to differing recommendations by different medical societies, a provider’s specialty may also impact their likelihood to recommend screening breast MRI.^31^ Patients may also be hesitant to undergo screening breast MRI due to concerns about insurance coverage and cost. One study found that 11% of women with *BRCA1/2* mutations were denied insurance coverage for an annual screening MRI from 2020 to 2021, with those insured through Medicaid experiencing the highest rate of denials.^32^

The underutilization of genetic counseling and testing has also been demonstrated by previous studies. For example, a study assessing referral for genetic evaluation among women at risk for breast and ovarian hereditary cancers found that only 15% of these patients had prior genetic testing recommended by their providers.^18^ While genetic testing is frequently recommended for women who have received a breast cancer diagnosis, there is a paucity of literature regarding the utilization of genetic testing among first-degree relatives of women with breast cancer. As such, our results serve as a benchmark for the utilization rate of genetic testing in a population of women likely at an elevated lifetime risk of breast cancer based on family history.

Several sociodemographic factors correlated with a greater likelihood of utilizing breast MRI and genetic testing in this study. Those married or living with a partner were nearly twice as likely as their unmarried counterparts to discuss genetic testing, highlighting the role of partner support in promoting health behaviors.^33,34^ Screening breast MRI, genetic testing rates, and discussions with providers regarding genetic test receipt in the future were more common among those who used the Internet to communicate with their physician’s office, suggesting that patients who are more technologically savvy may be more likely to pursue these tests, likely due to access to online educational information.

This study found that individuals identifying as Black as well as patients without insurance and those with Medicaid displayed decreased odds of having genetic test discussions with their health care providers; however, no difference was shown in the odds of these groups receiving a genetic test. It is possible that individuals may seek out genetic testing through external sources without a discussion with their health care provider, such as through widely available direct-to-consumer genetic testing, which patients may prefer over tests in a health care setting.^35^ Patients may feel that these direct-to-consumer tests provide clear information, and they are able to access them at a known cost without worrying about insurance coverage or unexpected medical fees.^35,36^ Furthermore, our study found that individuals who worried about medical bills had no difference in the odds of having a genetic test discussion but had lower odds of actually receiving a genetic test.^37,38^ This is consistent with prior studies showing financial concerns impacting patients’ care-seeking behaviors. Lastly, as expected, prior receipt of screening MRI was associated with higher odds of genetic testing discussion and receipt.

There are several limitations to this study. Women were included based on a family history of breast cancer in a first-degree relative by the age of 50 as a proxy for being higher-than-average risk for breast cancer because we were not able to calculate individual breast cancer risk due to a lack of data. However, using the Tyrer Cuzick risk assessment, these women are likely to have greater than 20% lifetime risk of breast cancer. Furthermore, due to the cross-sectional design of the NHIS survey, causal relationships cannot truly be established among any variables. In addition, the data collected is self-reported, which introduces the potential for biases. Despite this, 1 study suggests that self-reported utilization of preventive health services reported through the NHIS survey, such as mammograms for early breast cancer detection, is a reliable measure of use and aligns with reports from physicians and electronic medical records.^39^

## Conclusion

The findings of this cross-sectional analysis of the NHIS 2023 identified underutilization of screening breast MRI and genetic testing among women with a family history of breast cancer in a first-degree relative before age of 50. Future research should focus on interventions to improve utilization of screening breast MRI and genetic testing, such as provider education programs and health policy improvements to increase insurance coverage.

## Figures and Tables

**Table 1.** Sociodemographic Characteristics of the Sample

Characteristics	Unweighted frequency, *N* (%), (*N* = 920)	Weighted frequency, *N* (%), (*N* = 6 851 757)
Age		
25–39 years	132/920 (14.3)	1 211 974/6 851 757 (17.7)
40–64 years	400/920 (43.5)	3 430 210/6 851 757 (50.1)
≥65 years	388/920 (42.2)	2 209 573/6 851 757 (32.2)
Race		
Asian	21/883 (2.4)	199 883/6 486 458 (3.1)
Black	119/883 (13.5)	932 502/6 486 458 (14.4)
White	699/883 (79.2)	5 063 194/6 486 458 (78.1)
Others^a^	44/883 (4.9)	290 879/6 486 458 (4.4)
Ethnicity		
Hispanic or Latino	96/920 (10.4)	855 463/6 851 757 (12.5)
Non-Hispanic or Latino	824/920 (89.6)	5 996 294/6 851 757 (87.50)
Marital status		
Married or living with a partner	489/902 (54.2)	3 896 177/6 687 188 (58.3)
Not married/living with a partner	413/902 (45.8)	2 791 011/6 687 188 (41.7)
Languages spoken at home		
English	596/677 (88)	4 238 478/4 988 556 (85)
Others	81/677 (12)	750 078/4 988 556 (15)
Education level		
Less than a college education	616/918 (67)	4 845 686/6 836 782 (70.9)
College education or higher	302/918 (33)	1 991 096/6 836 782 (29.1)
Worked for a pay at a business during last week		
Yes	386/902 (42.8)	3 177 439/6 671 265 (47.6)
No	516/902 (57.2)	3 493 826/6 671 265 (52.4)
Health insurance		
Commercial	476/917 (51.9)	3 704 500/6 830 076 (54.2)
Medicare and other government-offered insurances (except for Medicaid)^b^	296/917 (32.3)	1 831 746/6 830 076 (26.8)
Medicaid or uninsured	145/917 (15.8)	1 293 830/6 830 076 (18.9)
Household family income to poverty threshold ratio		
0–1.00	122/920 (13.3)	857 677/6 851 757 (12.5)
1.01–2.00	196/920 (21.3)	1 411 691/6 851 757 (20.6)
≥2.00	602/920 (65.4)	4 582 389/6 851 757 (66.9)
Living region^c^		
Nonmetropolitan	155/920 (16.9)	1 036 985/6 851 757 (15.1)
Metropolitan	765/920 (83.1)	5 814 773/6 851 757 (84.9)
Worrying about medical bills in times of getting sick or accident	434/919 (47.2)	3 305 880/6 839 425 (48.3)
Using the Internet to communicate with doctor’s office	450/851 (52.9)	3 366 994/6 463 513 (52.1)
Number of family members with breast cancer		
1	651/920 (70.8)	4 858 777/6 851 757 (70.9)
2 or more	269/920 (29.2)	1 992 980/6 851 757 (29.1)
Time since the last doctor’s visit		
Within the last 2 years	886/920 (96.3)	6 563 074/6 851 757 (95.8)
More than 2 years ago	34/920 (3.7)	288 683/6 851 757 (4.2)
Having comorbidities	699/920 (76)	5 034 511/6 851 757 (73.5)

a American Indian, Alaska Native, Native Hawaiian, Pacific Islander, mixed, or other races.

b Children’s Health Insurance Program; military-related health care: TRICARE (Civilian Health and Medical Program of the Uniformed Services), Veterans Affairs health care, Civilian Health and Medical Program of Veterans Affairs; Indian Health Service, state-sponsored health plan, and other government programs.

c Based on the 2013 National Center for Health Statistics urban-rural classification scheme for countries.^23^

**Table 2.** Utilization Rate of Screening Mammography, Screening Breast MRI, and Genetic Testing

Outcomes	Unweighted frequency, *n* (%) (*N* = 920)	Weighted frequency, *n* (%) (*N* = 6 851 757)
Ever discussed the possibility of receiving genetic testing with a doctor	249/917 (27.1)	1 963 524/6 822 303 (28.8)
Genetic test performed for cancer risk assessment	153/917 (16.7)	1 209 406/6 830 229 (17.7)
Ever had a screening mammogram	716/880 (81.3)	5 023 422/6 459 173 (77.8)
Had a screening mammogram within the last 2 years	562/880 (63.9)	4 034 489/6 459 173 (62.5)
Ever had a screening breast MRI	68/876 (7.8)	506 672/6 417 453 (7.9)
Had a screening breast MRI within the last year	30/876 (3.4)	228 044/6 417 453 (3.6)

**Table 3.** Factors Associated With Receipt of Any Prior Screening Breast MRI

	Univariable analysis OR (95% CI)	Multivariable analysis OR (95% CI)^a^
Age		
25–39 years	Reference	Reference
40 to 64 years	2.35 (0.58–9.49)	2.26 (0.56–9.17)
≥65 years	1.44 (0.39–6.60)	1.08 (0.23–5. 07)
Race		
Asian	1.07 (0.18–6.22)	0.15 (0.01–1.43)
Black	1.09 (0.51–2.34)	0.79 (0.30–2.10)
White	Reference	Reference
Others^b^	1.09 (0.34–3.53)	1.42 (0.43–4.75)
Ethnicity		
Not Hispanic or Latino	Reference	Reference
Hispanic or Latino	**0.34 (0.12–0.96)**	0.82 (0.22–2.96)
Marital status		
Not married/living with a partner	Reference	Reference
Married/living with a partner	**1.89 (1.04–3.44)**	1.85 (0.98–3.50)
Languages spoken at home		
English	Reference	-
Others	0.52 (0.20–1.36)	-
Level of education		
Less than a college education	Reference	Reference
College education or higher	1.57 (0.88–2.77)	1.23 (0.62–2.46)
Worked for pay at a business during last week		
No	Reference	Reference
Yes	1.15 (0.64–2.08)	0.94 (0.46–1.92)
Health insurance		
Commercial	Reference	Reference
Medicare and other government-offered insurances (except for Medicaid)^c^	1.09 (0.58–2.03)	1.57 (0.73–3.34)
Medicaid or uninsured	0.70 (0.30–1.62)	0.76 (0.25–2.25)
Household family income to poverty threshold ratio		
0–1.00	Reference	-
1.01–2.00	0.43 (0.13–1.394)	-
≥2.00	0.95 (0.40–2.27)	-
Living region^d^		
Nonmetropolitan	Reference	-
Metropolitan	1.20 (0.45–3.20)	-
Number of family members with breast cancer		
1	Reference	Reference
2 or more	**1.91 (1.06–3.45)**	**2.94 (1.56–5.54)**
Time since the last doctor’s visit		
Within the last 2 years	Reference	-
More than 2 years	0.24 (0.03–1.86)	-
Having comorbidities	1.00 (0.49–2.04)	-
Worrying about medical bills in times of getting sick or accidents	0.87 (0.49–1.56)	0.84 (0.43–1.65)
Using the Internet to communicate with doctor’s office	**2.51 (1.37–4.59)**	**3.02 (1.53–6.00)**
Receipt of screening mammography in the last 2 years	**2.02 (1.01–4.04)**	1.78 (0.85–3.75)

Bolding represents *P*-value <.05.

Abbreviation: OR, odds ratio.

a Number of observations in multivariable analysis was 758 unweighted and 5 586 188 weighted.

b American Indian, Alaska Native, Native Hawaiian, Pacific Islander, mixed or other races.

c Children’s Health Insurance Program; military-related health care: TRICARE (Civilian Health and Medical Program of the Uniformed Services), Veterans Affairs health care, Civilian Health and Medical Program of Veterans Affairs, Indian Health Service, State-sponsored health plan, and other government programs.

d Based on the 2013 National Center for Health Statistics urban-rural classification scheme for countries.^23^

**Table 4.** Factors Associated With the Discussion of Possibly Receiving a Genetic Test With Their Doctor or Completing a Genetic Test

	Genetic test discussion OR (95% CI)		Genetic testing OR (95% CI)	
	Univariable	Multivariable^a^	Univariable	Multivariable^b^
Age				
25–39 years	Reference	Reference	Reference	Reference
40 to 64 years	**0.52 (0.33–0.82)**	**0.37 (0.20–0.67)**	**0.45 (0.27–0.76)**	**0.29 (0.15–0.56)**
≥65 years	**0.25 (0.15–0.42)**	**0.14 (0.06–0.33)**	**0.31 (0.18–0.53)**	**0.21 (0.08–0.55)**
Race				
Asian	1.90 (0.66–5.44)	1.67 (0.64–3.30)	2.10 (0.68–6.43)	1.84 (0.59–5.77)
Black	**0.52 (0.30–0.90)**	**0.43 (0.20–0.91)**	0.60 (0.30–1.16)	0.53 (0.23–1.21)
White	Reference	-	Reference	Reference
Others^c^	0.73 (0.33–1.61)	0.48 (0.15–1.57)	0.71 (0.26–1.94)	0.62 (0.13–2.91)
Ethnicity				
Not Hispanic or Latino	Reference	Reference	Reference	Reference
Hispanic or Latino	0.86 (0.49–1.50)	0.83 (0.35–1.98)	0.72 (0.36–1.47)	1.18 (0.47–3.25)
Marital status				
Not married/living with a partner	Reference	Reference	Reference	Reference
Married/living with a partner	**2.18 (1.56–3.04)**	**1.71 (1.14–2.56)**	**1.56 (1.04–2.34)**	1.34 (0.84–2.15)
Languages spoken at home				
English	Reference	-	Reference	-
Others	1.10 (0.60–2.00)	-	1.61 (0.83–3.14)	-
Level of education				
Less than a college education	Reference	Reference	Reference	Reference
College education or higher	**2.25 (1.60–3.16)**	1.45 (0.96–2.19)	**2.52 (1.67–3.79)**	1.60 (0.97–2.64)
Worked for a pay at a business during last week				
No	Reference	Reference	Reference	Reference
Yes	**1.56 (1.11–2.19)**	0.93 (0.57–1.52)	1.23 (0.81–1.85)	0.73 (0.42–1.28)
Health insurance				
Commercial	Reference	Reference	Reference	Reference
Medicare and other government-offered insurances (except for Medicaid)^d^	**0.55 (0.37–0.81)**	1.35 (0.77–2.46)	**0.46 (0.28–0.73)**	0.58 (0.27–1.27)
Medicaid or uninsured	**0.61 (0.38–0.99)**	**0.45 (0.23–0.87)**	0.63 (0.35–1.13)	0.59 (0.27–1.26)
Household family income to poverty threshold ratio				
0–1.00	Reference	-	Reference	-
1.01–2.00	0.73 (0.38–1.41)	-	0.66 (0.29–1.49)	-
≥2.00	1.54 (0.89–2.68)	-	1.45 (0.74–2.82)	-
Living region^e^				
Nonmetropolitan	Reference	-	Reference	-
Metropolitan	1.17 (0.74–1.87)	-	1.48 (0.83–2.63)	-
Number of family members with breast cancer				
One	Reference	Reference	Reference	Reference
Two or more	**1.85 (1.30–2.64)**	**2.62 (1.69–4.05)**	**1.95 (1.28–2.96)**	**2.74 (1.68–4.48)**
Time since the last doctor’s visit				
Within the last 2 years	Reference	Reference	Reference	Reference
More than 2 years ago	**0.32 (0.10–0.97)**	**0.18 (0.05–0.68)**	0.26 (0.05–1.15)	0.47 (0.12–1.83)
Having comorbidities	0.92 (0.62–1.36)	-	0.87 (0.54–1.39)	-
Worrying about medical bills in times of getting sick or accidents	1.03 (0.73–1.45)	1.07 (0.70–1.63)	**0.59 (0.39–0.91)**	**0.54 (0.33–0.90)**
Using the Internet to communicate with doctor’s office	**3.36 (2.32–4.86)**	**2.34 (1.51–3.62)**	**2.97 (1.90–4.64)**	**2.49 (1.48–4.20)**
Receipt of screening MRI	**5.75 (3.23–10.24)**	**5.60 (3.00–10.45)**	**4.48 (2.50–8.14)**	**4.04 (2.00–8.16)**

Bold represents *P*-value <.05.

Abbreviation: OR, odds ratio.

a Number of observations in multivariable analysis: 756 unweighted and 5 559 073 weighted.

b Number of observations in multivariable analysis: 756 unweighted and 5 566 999.

c American Indian, Alaska Native, Native Hawaiian, Pacific Islander, mixed or other races.

d Children’s Health Insurance Program; military-related health care: TRICARE (Civilian Health and Medical Program of the Uniformed Services), Veterans Affairs health care, Civilian Health and Medical Program of Veterans Affairs; Indian Health Service, State-sponsored health plan, and other government programs.

e Based on the 2013 National Center for Health Statistics urban-rural classification scheme for countries.^23^

## Key Messages

- In the 2023 National Health Interview Survey, only 7.9% of women with a first-degree relative diagnosed with breast cancer at age ≤50 had a screening breast MRI, and just 3.6% had done so in the past year.
- Genetic testing utilization was 17.7% among this group of high-risk women.
- Findings suggest underutilization of both tests, despite recommendations by national guidelines.
